# Tailored biosynthesis of medium chain oleochemicals by engineering fatty acid synthase and β-oxidation

**DOI:** 10.1016/j.synbio.2026.06.010

**Published:** 2026-07-29

**Authors:** Simin Cui, Jinming Gao, Xiaobing Yang

**Affiliations:** a College of Enology, Northwest A&F University, Yangling, Shaanxi, 712100, China; b Shaanxi Key Laboratory of Natural Products and Chemical Biology, Yangling, Xianyang, 712100, China

**Keywords:** Microbial production, Protein engineering, Limited β-oxidation, Short chain oleochemicals, Medium chain oleochemicals

Short-chain fatty acids (SCFAs) (≤C8) find their way in food, pharmaceutical and cosmetic industries while the medium-chain ones (MCFAs) (C10 –C14) are used as lubricants, fragrances, paint, etc [[1], [2], [3]]. Manufacturing short- and medium chain oleochemicals (S/MCOs) from coconut- and palm oils suffers from limited growing area, unpredictable climate hazards, and long planting cycle [4]. Microbial production of the versatile S/MCOs stands as a promising alternative, however, their titers lag far behind the industrial demands, and it is challenging in controlling the chain length for production of specific fatty acid (FA). Recently, an international collaboration between groups of Martin Grininger and Yongjin J. Zhou systematically engineered the metazoan FA synthase (mFAS) [5] and β-oxidation process to enable specific production of MCFAs in yeast [6].

In contrast to the enclosed rigid architecture in fungi, and the multi-subunit pattern in bacteria, the distinctive structure of mFAS permits a certain degree of fine-tuning while harbors considerable structural rigidity. The fatty acyl chain length is determined by a substrate-tolerant elongation process, kept by ketoacyl synthase (KS), β-ketoacyl reductase (KR), dehydratase (DH) and enoyl reductase (ER), and a substrate specific releasing thioesterase (TE). Common strategies for controlling the MCFAs chain length include: (i) modifying the fungal FAS volume or cavity size via multiple mutations and adaptive laboratory evolution; (ii) engineering the TE or KS domain independently; (iii) employing the reversed β-oxidation (r-BOX). Generally, the strategies mentioned above have poor product specificity. As a comparison, the advantages of the strategy developed by Martin Grininger and Yongjin J. Zhou groups lies in its achievable precise chain-length switching of the fatty acyl-CoAs, and the universality in enabling the production of medium chain fatty acids, aldehydes and alcohols. Furthermore, it exhibits robust host compatibility in *Ogataea polymorpha* and *Saccharomyces cerevisiae*. This study first shifted the product spectrum of mFAS to cover MCFAs (C12–C18) by replacing the native TE domain with the *Escherichia coli* derived ‘TesA [7] to demonstrate the plasticity of mFAS. Then, they managed to alter the substrate specificity of KS in mFAS, which determines the loading of the acyl chain to the active cysteine residue (Cys161 in murine mFAS) of KS and the bound acyl moiety elongation. By replacing sterically demanding residues (Gly113, Ser117, Met132, Ala160 and Thr196), the authors generated 14 binding tunnel mutants of the KS based on structural analysis of the KS-bound octanoyl moiety carrying murine mFAS as well as guided by the KSs from the hexanoyl FAS/PKS production systems. *In vitro* tests showed that the product spectra of the mutated mFAS shifted toward shorter acyl chains as the sterically demanding amino acid size increased. More interestingly, except for G113M, when the KS mutants were incorporated into the full mFAS, decreased activity and dropped FAs yields were observed. Subsequently, chain length modulation was attained using the hybrids of ‘TesA variants and the selected KS mutants. Of note, the hybrids, mFAS^G113S^/‘TesA^M141L/E142D/Y145G/L146K^ (‘TesA_4x) and mFAS^G113M^/‘TesA surpassed the wild mFAS rates, which can be attributed to the high elongation rates of G113S and G113M for short acyl chains that align perfectly with the acyl chain specificity of ‘TesA and ‘TesA_4x.

Replacing the TE domain with a fatty acyl-CoA reductase (TR) domain resulted in at least two orders of magnitude lower than the overall mFAS activity, which would impose a kinetic bottleneck on fatty alcohol/aldehyde production. To match the kinetics of KS and TR, combining the use of G113W mutation in KS and TRs^CAR^ [8] from *Mycobacterium phlei*, *M. smegmatis* or *Nocardia otitidiscaviarum*, enabled a 3-fold increase in enzyme activity and 8-fold improvement in fatty alcohol biosynthesis.

The other inspiring part of their work is deploying the production of S/MCFAs in yeast via rational β-oxidation. While β-oxidation is generally taken as a catabolic process and often blocked for enhancing S/MCFAs production, they demonstrated its rewirable potential for manipulating acyl chain length in the thermotolerant yeast *O. polymorpha* [9]. First, with lauric acid as the target (C12), they showcased the precise biosynthesis of MCFAs through both plasmid-based and genome-integrated expression of the three constructs mFAS^G113S^/‘TesA, mFAS^G113M^/‘TesA and mFAS^G113F^/‘TesA in *O. polymorpha*. Then, the C12 titer was substantially improved via either deleting the rate-limiting FA oxidase gene *POX1* or targeting the degradation of long-chain fatty acids (LCFAs) by replacing the native acyl-CoA ligase gene *OpFAA1* with the long chain preferring *S*. *cerevisiae ScFAA1.* Subsequently, for the design of limited β-oxidation to redirected the degradation of LCFAs toward MCFAs, they combined the genes overexpression of long-chain acyl-CoA oxidase (LAO) and medium-chain acyl-CoA releasing TE. Finally, MCFAs titer was elevated to 90.8 mg/L via co-expressing the LAO gene *YlPOX2* from *Yarrowia lipolytica* and human TE gene *hACOT4*, deleting the native FA oxidase gene *POX1*, and increasing the mFAS expression levels. Notably, the C12-FA accounted for 69% of the total free FAs fraction. Converting endogenous but undesired C16–C18 FAs into target MCFAs not only improved carbon utilization efficiency but also minimized the perturbation on cellular redox homeostasis. Further, fed-batch fermentation in the 1.5-L bioreactor yielded 709 mg/L MCFAs, consisting of 189.5 mg/L C10-FA, 484.1 mg/L C12-FA and 34.0 mg/L C14-FA. Importantly, the FA composition resembled that of the palm kernel oil.

In summary, a tailored strategy for manipulating the FA chain length has been developed (Fig. 1) that combining enzyme and metabolic engineering is a powerful strategy for specific biosynthesis of oleochemicals with precise chain-length control [10]. However, the current titer of S/MCFAs remains far below the commercialization threshold (>10 g/L). The low secretion efficiency poses another critical challenge as high intracellular content nullifies upstream fermentation advantages and elevates downstream purification costs. Furthermore, the long fermentation period also hinders the economic efficiency. To address the issues above, future endeavors include extensive the KS and TE domain engineering, precursor supply and NADPH balance, S/MCFAs secretion and fermentation system optimization should be implemented.

**Fig. 1:**
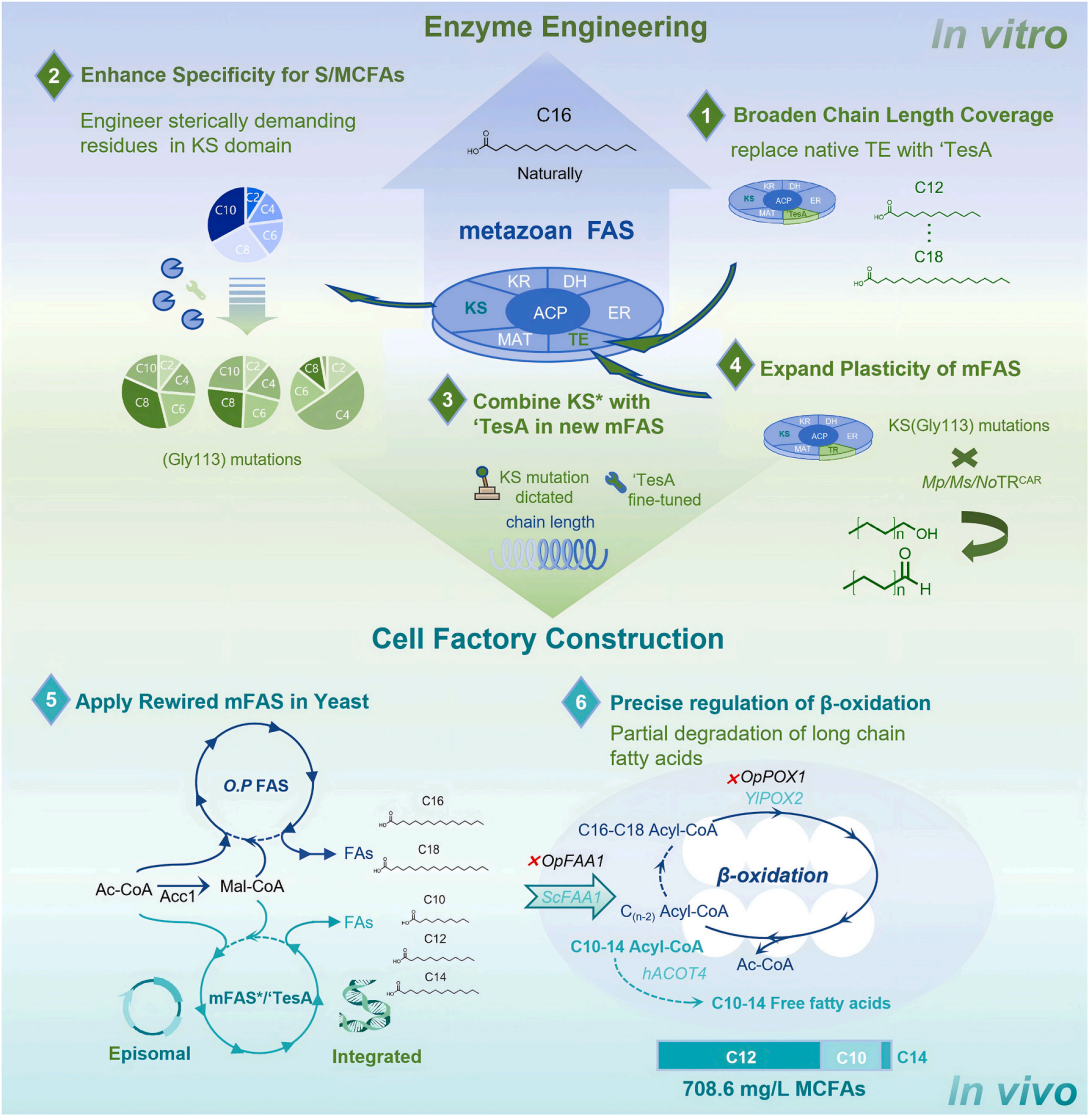
Strategies for tailored biosynthesis of medium chain fatty acids. Ac-CoA: acetyl-coenzyme A; Mal-CoA: malonyl-CoA; FAs: fatty acids; C_n_Acyl-CoA: fatty acid acyl-CoA, n = 18,16, …,4; MCFAs: medium chain fatty acids; FAS: fatty acids synthase; *O.P* FAS: fatty acids synthase of *O. polymorpha*; mFAS*/‘TesA: Metazoan FAS/‘TesA hybrids with different KS mutations; β-oxidation: the peroxisomal β-oxidation for fatty acids; ACP: acyl carrier protein; MAT: malonyl-acetyl transferase; KS: ketoacyl synthase; KR: ketoacyl reductase; DH: dehydratase; ER: enoyl reductase; TE: thioesterase; ‘TesA: truncated version of thioesterase TesA from *Escherichia coli*; TR: thioester reductase; *Mp/Ms/No*TR^CAR^: thioester reductase from *M. phlei, M. smegmatis* and *N. otitidiscaviarum*; *OpFAA1*: acyl-CoA ligase encoding gene of *O. polymorpha*; *ScFAA1*: acyl-CoA ligase encoding gene of *S. cerevisiae*; *OpPOX1*: FA oxidase gene in *O*. *polymorpha*; *YlPOX2*: FA oxidase gene from *Y. lipolytica*; *hACOT4*: human thioesterase encoding gene.

## CRediT authorship contribution statement

**Simin Cui:** Writing – original draft, Writing – review & editing. **Jinming Gao:** Conceptualization. **Xiaobing Yang:** Funding acquisition, Project administration, Writing – original draft, Writing – review & editing.

## Declaration of competing interest

The authors declare that they have no known competing financial interests or personal relationships that could have appeared to influence the work reported in this paper.
